# Ascertaining cause of mortality among middle-aged and older persons using computer-coded and expert review verbal autopsies in the China Health and Retirement Longitudinal Study

**DOI:** 10.1080/16549716.2020.1768502

**Published:** 2020-06-16

**Authors:** Yuan S. Zhang, Peifeng Hu, John A. Strauss, Yaohui Zhao, Yafeng Wang, Eileen M. Crimmins

**Affiliations:** a Carolina Population Center, University of North Carolina, Chapel Hill, NC, USA; b David Geffen School of Medicine, University of California, Los Angeles, CA, USA; c Department of Economics, University of Southern California, Los Angeles, CA, USA; d National School of Development, Peking University, Beijing, China; e Institute of Social Surveys, Peking University, Beijing, China; f School of Gerontology, University of Southern California, Los Angeles, CA, USA

**Keywords:** Verbal autopsy, cause of death, aging, China Health and Retirement Longitudinal Study (CHARLS), developing countries

## Abstract

**Background:**

Verbal autopsy is designed to ascertain causes of death that are not registered or certified. Verbal autopsy has been validated in multiple settings but has not been as widely evaluated for older populations as for younger age groups.

**Objective:**

This study aims to provide empirical evidence of the value of verbal autopsy interviews in the context of population-based surveys of older adults by comparing the cause-of-death assignments derived from two methods of interpreting verbal autopsy data.

**Methods:**

Data used in this study come from the China Health and Retirement Longitudinal Study, a nationally representative longitudinal survey of older Chinese. We compared 407 causes of death determined using InterVA, which is a computer-coded method, and causes of death as assigned by experts; then evaluated factors that affect the results of the two approaches.

**Results:**

Among the 407 deaths, neoplasms, cardiac disease, and stroke are the leading causes of death according to both approaches. The consistency of the two approaches is about 45% at the individual level. The primary reason for the mismatch is that no cause of death could be assigned for more than 25% of the sample based on expert review. A higher likelihood of mismatch is associated with advanced age and a long period between death and verbal autopsy interview.

**Conclusion:**

Both approaches identify the same leading causes of death at the aggregate level, but consistency is relatively low at the individual level. InterVA works well when causes of death are characterized by distinctive signs and symptoms. Grouping the various causes of death with shared etiology or common risk factors may help improve the quality of the ascertainment of causes of death. Open-ended narratives are helpful because they provide information about the circumstances surrounding the death that are not available in the structured verbal autopsy interviews.

## Background

The study of cause-specific mortality is crucial for understanding the reasons that mortality risks differ across populations or subgroups. However, assigning a cause of death is challenging when deaths occur outside of health facilities and are not attended by qualified medical personnel. This challenge is particularly relevant for low- and middle-income countries where most deaths occur at home, and vital statistics systems cannot provide reliable cause-specific mortality [1]. Globally, about one-third of all countries are capable of producing high-quality cause of death data, and most of these countries are high-income countries in Europe and North America [2]. More than 140 other countries, representing 80% of the world’s population, either produce lower quality cause of death data or lack such data [2]. Verbal autopsy is designed to determine causes of death by collecting information from persons who were close to the deceased in their final illness or time before death [3]. Applying verbal autopsy in the context of population-based surveys may allow researchers to determine causes of death from individual survey respondents and link causes to risk factors such as early-life experiences, socioeconomic conditions, health behaviors, and disease history.

In recent decades, the World Health Organization (WHO) has led an international effort to develop and improve standardized instruments, procedures of data collection, and approaches to cause of death assignments for verbal autopsy. The WHO introduced the first international technical standards and guidelines for verbal autopsy in 2007 [4]. The standard instrument included three sets of questionnaires that correspond to death of a child under 4-weeks old, death of a child aged 4 weeks to 14 years, and deaths of adolescents and adults (aged 15 years and above). A revised shortened instrument was published in 2012 and updated in 2014 and 2016 [5–7]. This simplified instrument contains questions that allow for responses with a simple yes/no answer or a duration in some instances. Either a computer-coded approach or physician review is then used to assign causes of death. Researchers have applied and validated multiple automated diagnostic and expert review methods to assign causes of death using verbal autopsy data for different age groups in many low-resource settings [8–14]. Evidence indicates that verbal autopsy provides relatively valid and reliable estimates of causes of pregnancy-related death [15], neonatal death [16], pneumonia [17], HIV/AIDS [18], adult non-communicable disease (NCD) mortality [19], and external causes of death [20,21]. Many studies have focused on the value of verbal autopsy in determining causes of death from infectious diseases and the causes of childhood and maternal deaths in areas such as Africa and South Asia where such problems are still major public health concerns. A few studies have applied verbal autopsy to adult mortality [8,19,22]. Nonetheless, the value of verbal autopsy for older populations in which chronic diseases are the major causes of death has not been studied as widely as for younger age groups. Classifying deaths for older adults poses many different issues. First, the potential causes of adult deaths are numerous in contrast to the limited number of causes for childhood deaths and maternal deaths [23]. Second, some chronic diseases are difficult to diagnose even in clinical settings [14]. Even with the use of advanced medical care, determining the underlying cause of death among very old and frail persons who often suffer from multimorbidity is challenging. Despite the challenges, however, better understanding the value of verbal autopsy in older populations has implications for developing improved cause of death data in the developing world, which is experiencing fast population aging [9]. Many of these countries have conducted surveys of older populations with extensive information about risk factors for health outcomes collected before deaths and interviews after death where cause of death would provide important information on health at the end of life.

This paper aims to address the use of verbal autopsy in a nationally representative sample of the older Chinese population by comparing the causes of death derived from two commonly used approaches: InterVA, a computer-based model that provides the probabilistic ascertainment of causes of death, and expert (physician) review. Although a number of automated diagnostic methods are available, we chose to use InterVA in this study because, to our knowledge, InterVA is the most frequently used automated model for interpreting verbal autopsy data, and has been validated in many settings [24].

Verbal autopsy results can be influenced significantly by instruments, characteristics of respondents, recall periods, and the methods used to interpret the data [25]. The instrument developed by the WHO uses a combination of open-ended questions that ask about the illness/events leading to the death and a series of closed-ended questions that ask whether specific symptoms and signs were present [5]. The computer-coded approach is faster and cheaper than physician review and improves inter-observer consistency and comparability [26,27]; however, it uses only closed-question data that may not adequately capture details about the chronic conditions of the deceased. Physician reviews usually incorporate written narrative text along with responses to closed ended questions in order to ascertain the cause of death. Such open-ended narratives from so-called ‘informants’ (people familiar with the circumstances of the deceased) may be particularly useful when they are combined with information from hospital reports and are able to detail other circumstances surrounding the death. ‘Informants’, i.e. verbal autopsy interview respondents, should be the primary caregiver who was with the deceased in the final period leading to death. However, the respondent may not have complete information about the health of the deceased. When verbal autopsy is applied in longitudinal household surveys, critical information about the health of the deceased that was provided by the deceased prior to his/her death can be incorporated to produce improved cause of death ascertainment. Ideally, verbal autopsy interviews should be conducted shortly after death occurs, as a long recall period is likely to have adverse impact on the informant’s ability to accurately report relevant information [28]. In this study, we combined data obtained from verbal autopsy interviews and baseline surveys of the respondents. We compared the causes of death derived from InterVA with assignments by experts and evaluated how characteristics of the deceased, verbal autopsy respondents, and verbal autopsy interviews affected the comparison of the two approaches. The results of this study are important for understanding the value of verbal autopsy interviews and improving the quality of verbal autopsy interviews in the context of population-based surveys.

### Causes of old-age mortality in China

China has gone through an epidemiological transition that has resulted in chronic diseases becoming the leading causes of death, with mortality increasingly concentrated in older populations. The vital registration system in China covers only selected regions, with data quality being better in developed areas (i.e. urban and eastern China) [29]. A validation study that compared causes of death based on expert reviews of medical records with causes of death that were filed in the registration system suggests that even death data from health facilities in urban China are prone to misclassification [15]. Wang et al. used data from 14 Disease Surveillance Points in rural China to compare the causes of death that were identified by verbal autopsy with diagnoses reported in registration data and found that verbal autopsy can substantially reduce the number of ill-defined causes of death, but the agreement between verbal autopsy and registration data was not high [30]. Yang et al. conducted a validation study using 3,290 deaths from six cities in urban China [14]. Their results suggest that verbal autopsy performed well for detecting deaths from stroke, cancer, and transportation, but was less reliable for ascertaining deaths that were due to heart disease, chronic pulmonary disease, diabetes, and kidney disease. However, the generalizability of the results was limited by the criteria used for sample selection. In the Yang et al. study, the deceased had to have been a resident of the city and the death had to have occurred in a tertiary care health facility. However, the performance of verbal autopsy would be worse when deaths occurred at home and in rural areas because families would have less information when the death occurred without interaction with the medical care system.

## Data and methods

The data used in this study came from the baseline survey of the China Health and Retirement Longitudinal Study (CHARLS) conducted in 2011–2012 and the CHARLS verbal autopsy interviews conducted in 2013. CHARLS is a nationally representative longitudinal survey of Chinese residents aged 45 and older, conducted by Peking University [31]. The national baseline survey interviewed 17,708 individuals; 441 of these individuals died before the follow-up interview in 2013. Verbal autopsy interviews were obtained for 407 deaths.

Respondents to the CHARLS verbal autopsy interviews were primarily spouses, children, or relatives of the deceased. CHARLS interviewers administered the 2012 WHO verbal autopsy instrument, which was translated into Chinese by the CHARLS team and then back-translated to English by staff at the China Center for Disease Control and Prevention (China CDC) to ensure accuracy. Before adopting the verbal autopsy instrument, the China CDC conducted a pretest of the Chinese verbal autopsy questionnaire for 14 deaths of persons 50 or older and compared the causes of death determined by verbal autopsy with those determined by China CDC experts based on clinical information and the interviews. The pretest results showed a match of cause of death in 57% of deaths. No evidence suggests that the discrepancies were due to questionnaire translation.

The CHARLS verbal autopsy instrument consists of both closed-ended questions and open-ended sections. The closed-ended questions include questions regarding previously known medical conditions, such as whether the deceased had any diagnosis of tuberculosis, malaria, hypertension, diabetes, cancer, stroke, asthma, chronic obstructive pulmonary disease (COPD), or kidney diseases, and symptoms noted during the final illness, such as whether the deceased had a fever, breathing problems, or diarrhea, history of injuries and accidents, as well as treatments and health service use during the period of final illness. Respondents also were asked to provide the three most likely causes of death as part of the verbal autopsy. More than half of the informants provided names of diseases that led to mortality based on their knowledge (e.g. ‘died of esophageal cancer’). About a quarter described the symptoms and circumstances preceding death in detail (e.g. ‘diagnosed with hypertension, had stroke 6–7 years ago, had cerebral hemorrhage and died’). About 20% could not provide meaningful details about deaths (e.g. ‘old age’, ‘no particular reason’, ‘died peacefully’).

In addition to the informants’ responses to questions regarding the presence of diseases reported in the verbal autopsy interviews, we incorporated information about prior diagnoses of diseases reported by respondents in the earlier household survey. If the deceased individual had reported having been diagnosed with hypertension, diabetes, cancer, asthma, COPD, stroke, kidney disease, or liver disease at the time of the household survey, we coded him/her as having this disease even if the respondent to the verbal autopsy interview did not report this. Only the responses to closed-ended questions were used as the input for InterVA. We processed the data using InterVA5 (Version 5.01) [24]. InterVA5 builds on the previous version, the InterVA4 model, and incorporates backward compatibility with the WHO 2012 verbal autopsy instrument and InterVA4 [32]. InterVA5 requires presetting two basic epidemiological parameters, the prevalence of HIV/AIDS and malaria; both were coded as very low for our application.

Four of the authors of this paper conducted the expert reviews. Our expert review panel included a Chinese-speaking physician who is board-certified as a geriatrician in the U.S. and experienced in assigning cause of death for older persons, two internationally recognized research scientists of health and aging, and a Chinese-speaking gerontologist. These four experts reviewed and discussed the open-ended narratives, the responses to the closed-ended verbal autopsy questions, the health conditions reported in the baseline household interview, and the verbal autopsy assigned causes of death for all cases together. We then assigned a likely cause for each case. The physician made the final decision in (rare) cases of disagreement. We aggregated the causes of death into the WHO simplified cause of death list [33]. Because the number of deaths that were due to nutritional and endocrine disorders, gastrointestinal disorders, and renal disorders was small, we grouped them into ‘Other NCDs’. We then used the three most likely causes of death and the propensity for each cause that was identified by InterVA5 to derive the cause-specific mortality fractions (CSMFs) [32], and compared the CSMFs from InterVA5 and the distribution of the causes of death determined from expert review. At the individual level, we compare the cause of death assignments assigned by InterVA5 and by expert review. We used the most likely cause assigned by InterVA5. Based on this comparison, we conducted logistic regression to examine how characteristics of the deceased, verbal autopsy respondents, and verbal autopsy interviews affected the comparison of the two approaches. To examine whether urban and rural populations differed in their responses to the verbal autopsy interviews and how this difference might affect the ascertainment, we classified the deceased based on their usual place of residence and their official household registration (hukou) when they were alive. Three categories were created: rural residency, urban residency and rural hukou, urban residency and urban hukou. The small percentage (7 out of 407, <2%) who lived in rural areas but had urban hukou were classified with other rural residents. This approach served to separate those who were living in urban areas by their ability to use medical services and the availability of those services.

## Results

The sample characteristics are presented in Table 1. The mean age at death was 72.5, with a standard deviation of 10.9 years. Females comprised 44.0% of the deaths. Most of the deceased had not completed primary school (64.4%); 63.6% lived in a rural area, 17.7% lived in an urban area but still had a rural hukou, and 18.7% lived in an urban area and had an urban hukou. Overall, most of the deceased died at home (82.1%). The percentage who died in hospitals was lowest among rural residents (8.1%) and highest among urban residents with urban *hukou* (42.1%), showing a clear rural-urban gradient. Of the verbal autopsy interviews, about 75% were provided by the child or spouse of the deceased; 28.5% were conducted within 6 months after death, 25.8% between 6 months and a year after death, and 27.8% between 13 and 18 months after death. We also examined the demographic characteristics of the 34 cases that were missing verbal autopsy interviews. The mean age at the baseline survey was 68.8; 15 participants (44.1%) were females; 56% lived in urban China before they died; 53% did not have formal education, 23.5% completed primary school, and 23.5% had junior school or higher education. Compared to those in the analytic sample, these 34 deceased missing verbal autopsy interviews were more likely to be urban residents and had more education.

**Table 1.** Characteristics of the Deceased in China Health and Retirement Longitudinal Study, 2011–2013, N = 407.

			Urban residency		
	All	Rural residency	Rural hukou	Urban hukou	
	N = 407	N = 259 63.6%	N = 72 17.7%	N = 76 18.7%	Chi-square test
Age at death, Mean (SD)	72.5 (10.9)	72.7 (10.7)	72.6 (10.8)	71.5 (11.6)	
Female, %	44.0	43.2	56.9	34.2	p = 0.019
Education of the deceased, %					p < 0.001
No formal schooling	64.4	71.4	73.6	31.6	
Primary school	18.4	17.0	16.7	25.0	
Junior/Secondary +	16.7	10.8	9.7	43.4	
Missing	0.5	0.8			
Place of death, %					p < 0.001
Hospital	15.2	8.1	12.5	42.1	
Home	82.1	90.7	80.6	54.0	
Other places	2.7	1.2	6.9	4.0	
Relationship of respondents to the deceased, %					p = 0.268
Children	34.9	37.5	34.7	26.3	
Spouse	39.8	36.3	41.7	50.0	
Others	25.3	26.3	23.6	23.7	
Time interval between death and VA interview, %					p = 0.433
0–6 months	28.7	29.3	22.2	32.9	
7–12 months	25.8	23.9	36.1	22.4	
13–18 months	27.8	27.8	26.4	29.0	
More than 18 months	17.7	18.9	15.3	15.8	

The CSMFs from InterVA5 and the distribution of the causes of death determined by expert review are shown in Table 2. Both approaches indicate neoplasms as the most frequent cause of death (26.9% for InterVA5 diagnosis and 24.1% based on expert review). InterVA5 classified 20.7% of deaths as due to stroke, which is about 5% higher than by expert review (16.2%). Both methods suggest that about 17% to 19% of deaths were due to cardiac diseases. Deaths due to infectious diseases, COPD, and external causes were low according to both methods (about 4%). Given our relatively small sample size, these two approaches generated fairly consistent distributions of cause of death at the aggregate level, especially for the causes mentioned here. Expert review could not determine a likely cause for more than a quarter of deaths in this sample after reviewing the written narrative text and responses to the standard closed-ended verbal autopsy questions. The percentage of cases without a cause assigned by InterVA5 was much lower, only about 12%.

**Table 2.** Cause-specific mortality fractions (CSMFs) derived from InterVA5 and Percentages of Causes of Death Determined by Expert Review, China Health and Retirement Longitudinal Study, 2011–2013, N = 407.

	InterVA5	Expert review
**Infectious**	**3.4**	**3.2**
Acute respiratory infection, including pneumonia	1.1	1.7
HIV/ADIS related death	0.4	
Pulmonary tuberculosis	1.9	1.2
Other and unspecified infectious disease		0.3
**Neoplasms**	**26.9**	**24.1**
Oral neoplasms	0.2	1.2
Digestive neoplasms	8.5	9.8
Respiratory neoplasms	8.8	5.4
Breast neoplasms	0.1	0.7
Reproductive neoplasms	4.6	0.5
Other and unspecified neoplasms	4.7	6.4
**Stroke**	**20.7**	**16.2**
**Diseases of the circulatory system, excluding stroke**	**19.2**	**16.7**
Acute cardiac disease	8.4	5.9
Other and unspecified cardiac diseases	10.8	10.8
**Respiratory disorders**	**4.4**	**3.7**
Chronic obstructive pulmonary disease	3.8	2.5
Asthma	0.6	1.2
**Other NCDs**	**9.5**	**4.9**
Nutritional and endocrine disorders	4.3	2.7
Gastrointestinal disorders	2.7	1.7
Renal disorders	1.9	0.5
Epilepsy	0.6	
**External causes of death**	**3.9**	**3.2**
**Indeterminate**	**12.0**	**28.0**

The bold emphases are for the major categories.

Table 3 presents a comparison of individual causes of death assigned by InterVA5 and by expert review. For the individual-level comparison, we used the most likely cause derived from the InterVA5. The highest levels of matching were for neoplasms and external causes of death (60.7% and 55.6%, respectively). Among the 122 deaths identified as neoplasms by InterVA5, 74 also were identified as such by expert review, and 28 deaths could not be assigned a cause by our research team. Among the 88 stroke deaths identified by InterVA5, 45 were also classified as stroke by expert review. Among the 89 cardiac disease deaths according to InterVA5, 36 were classified as cardiac disease, 8 as stroke, and 10 as neoplasms by expert review, whereas expert review could not determine a cause in 24 cases. The lowest levels of concordance were found for infectious and parasitic diseases and other NCDs. Among the 45 deaths determined by InterVA5 as due to other NCDs, only 3 of these deaths were classified as such by expert review; causes of death for 12 cases were identified as cardiac disease based on expert review, and the cause for 17 deaths could not be determined because the families/relatives of the deceased did not provide explicit and meaningful information about the deaths. This was also true for infectious and parasitic diseases and respiratory disorders that exhibited low levels of matching. With regard to the 114 deaths for which expert review failed to determine a likely cause, InterVA5 provided the three most frequent causes of death for 70% of these cases: 25% (28 cases) as neoplasms, 22% (24 cases) as cardiac disease, and 22% (24 cases) as stroke. We calculated kappa statistics to examine agreement between causes of death assigned by InterVA5 and by expert review. The negative value (−0.04) indicates no agreement. The percentage matching reported in the bottom of the tables shows that among the 98 cases identified as neoplasms deaths by expert review, InterVA5 identified 74 of them as such. Relatively high concordance is also found for external causes of death, stroke, and cardiac diseases. If expert review can assign a cause, it is very unlikely that InterVA5 fails to determine a cause.

**Table 3.** Differential Classification Matrix for Major Causes of Death, China Health and Retirement Longitudinal Study, 2011–2013.

Expert review											
InterVA5 assignment (most likely cause)	Infectious	Neoplasms	Stroke	Cardiac disease	Respiratory disorders	Others NCD*	External causes	Indeterminate	Total	% Matching	% Indeterminate
Infectious	**1**	1	1	3	4	1	0	4	15	6.7	26.7
Neoplasms	4	**74**	3	4	1	8	0	28	122	60.7	23.0
Stroke	2	3	**45**	8	1	3	2	24	88	51.1	27.3
Cardiac disease	3	10	8	**36**	3	5	0	24	89	40.4	27.0
Respiratory disorders	3	2	1	3	**5**	0	1	4	19	26.3	21.1
Others NCDs*	0	7	6	12	0	**3**	0	17	45	6.7	37.8
External causes	0	0	2	0	0	0	**10**	6	18	55.6	33.3
Indeterminate	0	1	0	2	1	0	0	**7**	11.0	63.6	/
Total	13	98	66	68	15	20	13	114	407		
% Matching	7.7	75.5	68.2	52.9	33.3	15.0	76.9	6.1			
% Indeterminate	0.0	1.0	0.0	2.9	6.7	0.0	0.0	6.1			

Other NCDs include nutritional and endocrine disorders, gastrointestinal disorders, and renal disorders. The bold numbers highlight the number of matched cases.

Based on the results presented in Table 3, we divided the sample into matched and unmatched groups. Table 4 reports the results from a multivariate logistic regression model predicting mismatch between the two approaches at the individual level. Deaths that occurred over the age of 80 were more than twice as likely to be diagnosed with a different cause with the two methods compared to deaths before age 70. An extended period between death and verbal autopsy interview, especially more than 18 months, is associated with a higher likelihood of mismatch.

**Table 4.** Odds Ratios from the Multivariate Logistic Model Predicting the Mismatch between the Diagnosis of InterVA5 and Expert Review.

	Odds ratio	95% CI
Age (ref = less than 70 years)		
70–79	1.5	(0.9, 2.6)
80+	2.2	(1.2, 4.0)
Female (ref = male)	1.4	(0.9, 2.3)
Urban-Rural (ref = rural residency)		
Urban residency, rural hukou	0.8	(0.4, 1.4)
Urban residency, urban hukou	0.7	(0.4, 1.3)
Education (ref = no formal schooling)		
Primary school	0.7	(0.4, 1.2)
Junior school or above	1.4	(0.7, 2.7)
Place of death (ref = hospital)		
Home	1.1	(0.6, 2.1)
Other	0.4	(0.1, 1.6)
Relationship to the deceased (ref = children)		
Spouses	0.7	(0.4, 1.1)
Other	1.0	(0.6, 1.7)
Time duration between death and VA interview (ref = 0–6 months)		
7–12 months	1.7	(0.9, 3.0)
13–18 months	1.1	(0.6, 2.0)
More than 18 months	2.0	(1.1, 3.7)
Unexpected death (ref = expected)	1.1	(0.7, 1.6)
Number of Observations	407	
Log likelihood	−259.9	(Prob > chi2 = 0.001)
Pseudo R2	0.1	

## Conclusions

This study used data from the CHARLS verbal autopsy interviews and baseline survey to ascertain causes of death in a sample that is representative of the older Chinese population. The representativeness of the sample ensures the generalizability of the results to China as a whole. Neoplasms, cardiac disease, and stroke were the most frequent causes of death determined by InterVA and expert review, which accounts for about 60% to 70% of total deaths. For these three major causes, the percentage matching between the two approaches was about 60% for neoplasms and about 40% to 50% for both stroke and cardiac disease. The main reason for the mismatch is that, with expert review, no cause was assigned for more than a quarter of the sample. In contrast, InterVA5 assigned 70% of the indeterminate causes as one of the three most frequent causes, namely neoplasms, cardiac disease, and stroke. A greater likelihood of mismatch is associated with advanced age and deaths that occurred at home. As mentioned, verbal autopsy is designed to assign a cause for deaths that occur at home when the cause of death cannot be certified by qualified medical personnel, but we are less confident about the accuracy of the assignments in cases where the informants were unable to provide sufficient relevant details. Both approaches that are used to assign causes of death appear to perform well when the cause of death can be characterized by distinctive signs and symptoms that do not overlap with other causes, such as neoplasms and external causes of death.

Ascertaining causes of death in older populations has specific challenges. Older adults often suffer from multiple life-threatening conditions at the time of death. Many chronic conditions have similar risk factors and share common signs and symptoms. For instance, breathlessness is a symptom of many possible causes of death, including COPD, lung cancer, pneumonia, and congestive heart failure. The common risk factors make the identification of a single most likely cause for each death a challenge. Although the assignment of multiple causes requires further methodological thinking, a feasible approach is to use the ‘broad category’ method [14,25]. If causes of death with shared etiology or common risk factors are considered together, the agreement will be enhanced and the accuracy of the assignment will be improved.

Validation studies typically use clinical diagnoses as the gold standard [12,14,17]. In this study, the absence of a reference standard made it impossible to compare the results to a gold standard. Because no age-specific cause of death data have been published by the Chinese government, we could not compare the distributions of causes of death in our study to official statistics. However, high levels of agreement between the results of the two approaches provide some confidence in cause of death assignments obtained from verbal autopsies for this population. Detailed reviews of the narrative sections that were included with the verbal autopsy instrument provided significant evidence for cause of death ascertainment for many sample members. As more people use medical care and die in hospitals, informants should be increasingly better able to provide details in surveys that can lead to better assignment of cause of death.

This study had some limitations. First, the lack of a gold standard made it impossible to conclude the relative accuracy of the two approaches. Second, the sample size was relatively small, particularly for rare causes, as only two-year mortality was assessed. A re-examination of the results when more deaths are observed over time would be worthwhile. Because of the design of our longitudinal study, the verbal autopsy interviews were not used immediately after the deaths occurred. As noted, the verbal autopsy interviews for these 407 cases were all conducted in the summer of 2013. Thus, the relatively long recall periods could threaten the accuracy of the information provided in the verbal autopsy interviews. In addition, InterVA5 is only one of several verbal autopsy algorithms that can be used to assign causes of death. The literature that describes the performance of different algorithms has not reached a consensus on which algorithms perform best or even how to conduct such comparisons [34]. We chose to use InterVA because it has been developed and modified over many years, and has been widely used in many settings. A comparison of different automated methods for the cause of death assignment is beyond the scope of this paper.

Despite the limitations, our study ascertains the causes of death in a nationally representative survey of the older population. Our analysis found that InterVA and expert review identify the same leading causes with similar percentages at the aggregate level, providing confidence regarding the accuracy in assessing a CSFM using InterVA. However, individual evaluations may be required to assign more accurate causes of death at the individual level. While many longitudinal surveys are collecting verbal autopsy data in the developing world, the results of this study provide important empirical evidence of the value of conducting verbal autopsy interviews in the context of population-based surveys of older adults.

## Paper context

This study addresses the value of including verbal autopsy in population-based surveys of older persons in which chronic diseases are the major causes. We compared the causes derived from a computer-coded approach with the assignments by experts. Two approaches yield same leading causes at the aggregate level, but the consistency is poor at the individual level. Grouping causes with shared etiology or common risk factors would help improve the quality of ascertainment.
